# The trans-kingdom identification of negative regulators of pathogen hypervirulence

**DOI:** 10.1093/femsre/fuv042

**Published:** 2015-10-13

**Authors:** Neil A. Brown, Martin Urban, Kim E. Hammond-Kosack

**Affiliations:** Department of Plant Biology and Crop Science, Rothamsted Research, Harpenden, Herts AL5 2JQ, UK

**Keywords:** pathogen, bacteria, fungi, virulence, emerging infectious diseases, antimicrobial resistance

## Abstract

Modern society and global ecosystems are increasingly under threat from pathogens, which cause a plethora of human, animal, invertebrate and plant diseases. Of increasing concern is the trans-kingdom tendency for increased pathogen virulence that is beginning to emerge in natural, clinical and agricultural settings. The study of pathogenicity has revealed multiple examples of convergently evolved virulence mechanisms. Originally described as rare, but increasingly common, are interactions where a single gene deletion in a pathogenic species causes hypervirulence. This review utilised the pathogen–host interaction database (www.PHI-base.org) to identify 112 hypervirulent mutations from 37 pathogen species, and subsequently interrogates the trans-kingdom, conserved, molecular, biochemical and cellular themes that cause hypervirulence. This study investigates 22 animal and 15 plant pathogens including 17 bacterial and 17 fungal species. Finally, the evolutionary significance and trans-kingdom requirement for negative regulators of hypervirulence and the implication of pathogen hypervirulence and emerging infectious diseases on society are discussed.

## Abstract (precis)

This trans-kingdom review of pathogenesis identifies the conserved molecular themes behind hypervirulence, and discusses the evolutionary significance for negative regulators of hypervirulence in animal and plant hosts and their implication on society.

## THE PHENOMENON OF PATHOGEN HYPERVIRULENCE

Modern society and global ecosystems are increasingly under threat from bacterial, protozoan, fungal and nematode pathogens, which cause a plethora of human, animal, invertebrate and plant diseases (Furuya and Lowy 2006; Nordmann, Naas and Fortineau 2007; Brown *et al.*^2012^; Fisher *et al.*^2012^; Jones *et al.*^2013^; Vanaerschot *et al.*^2013^). A trans-kingdom tendency for increased pathogen virulence (hypervirulence) is beginning to emerge and be noted. The genetic flexibility of pathogens, most of which have haploid genomes, permits rapid evolutionary changes, which can result in altered phenotypes such as host species range, environmental range, stress tolerance, increased virulence and/ or antimicrobial resistance. ‘Superbugs’ and ‘superpests’ are causing issues in our hospitals and farms because of increasing drug resistance (Nordmann, Naas and Fortineau 2007; Andersson and Hughes 2010). This cohort of invasive bacterial and fungal infections of humans and animals are notoriously difficult to treat and cause unacceptably high disease and mortality rates (Furuya and Lowy 2006; Brown *et al.*^2012^). At the same time, highly virulent fungal and protist pathogens of wild animals have recently emerged and are decimating amphibian and bat populations worldwide, with unknown consequences on these natural ecosystems (Fisher *et al.*^2012^). Plants are the most important provider of human calories and a major influence on human health. Severe disease epidemics in any of the major crops would have a substantial impact on global food security and human health (Fisher *et al.*^2012^; Jones *et al.*^2013^). Similarly, parasitic nematodes of humans cause diseases that have a major impact on human health, while also causing yield losses in agriculture systems, including domesticated animals and crops, exacerbating the global food shortage (Jones *et al.*^2013^).

**Figure 1.**
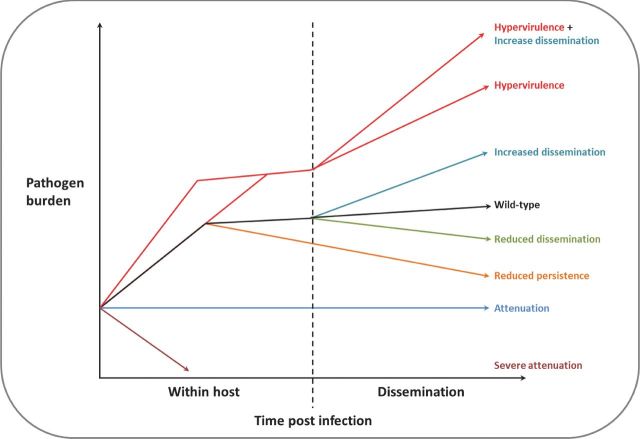
The different ways in which hypervirulence and increased pathogen dissemination can contribute to disease outbreaks. Presented is the virulence phenotype of wild-type and mutant pathogens within the host organism and subsequently in the environment post dissemination (adapted from Ten Bokum *et al.*^2008^). Hypervirulent mutants multiply at the same rate, or faster, within the host, reaching a higher final pathogen burden. Mutants with an increased capacity to be disseminated beyond the host can also cause a rise in pathogen burden, transmission and disease incidence, even when in a wild-type pathogenic background. The combination of hypervirulence and increased dissemination poses the greatest threat to the host organism. In contrast, mutants with reduced dissemination capacity, reduced persistence or attenuated virulence will result in a decline in pathogen burden.

Multiple examples of trans-kingdom, convergently, evolved virulence mechanisms exist. Bacterial, protozoan, fungal, protist and nematode pathogens secrete toxic and non-toxic secondary metabolites and proteins into their respective host organisms to either manipulate host responses or to induce cell death, potentiating disease (Engel and Balachandran 2009; Jones *et al.*^2013^; Wirthmueller, Maqbool and Banfield 2013; Brown and Hammond-Kosack 2015). Modern pathogenomics (a whole genome approach to the study of pathogenesis), predominantly through the use of small- to large-scale reverse genetics, has increasingly facilitated the dissection of the genetic elements that determine virulence, following the assumption that the loss of a gene involved in pathogenesis will be detrimental to virulence. However, originally described as rare, but increasing common, are interactions where single gene deletions cause hypervirulence. The phenomenon of hypervirulence is defined as pathogenic strains which (i) multiply at a faster rate than the wild-type strain during the initial phase of innate immunity; (ii) stabilise at a higher level of pathogen burden, or continues to multiply at a faster rate, than the wild-type strain, after the initiation of the adaptive immune response; and (iii) may have a greater potentially for dispersal (Fig. 1) (ten Bokum *et al.*^2008^). The current scientific understanding of the natural rise of hypervirulent clinical, agricultural or environmental pathogens is hampered by the detection of numerous genetic differences and pathogen lifestyles that may contribute to the altered virulence profile. Hence, the wide scale identification and functional characterisation of single genetic elements that result in hypervirulence would greatly enhance our understanding of the costs and benefits that could arise when individuals within a pathogen population possess the ability to cause a greater disease load.

## THE PATHOGEN–HOST INTERACTION DATABASE (PHI-base)

**Figure 2.**
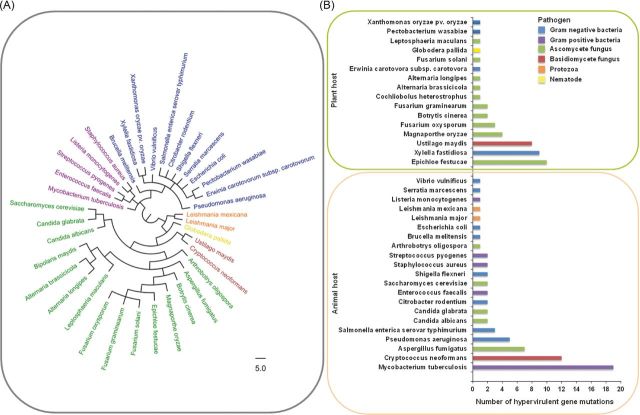
The trans-kingdom distribution of 112 negative regulators of pathogen hypervirulence. (**A**) The phylogenetic distribution of the negative regulators of hypervirulence, identified within the pathogen–host interactions database (PHI-base), including bacterial, protozoan, fungal and nematode pathogens. The tree was generated using the NCBI taxonomy tree building service (Sayers *et al.*^2009^). (**B**) The number of hypervirulent mutations per pathogenic species subdivided into animal (n = 67) and plant (n = 45) attacking pathogens.

Over the past decade, PHI-base (www.PHI-base.org) has accumulated ∼5000 curated interactions from the peer-reviewed literature published since 1987, providing open access phenotypic and molecular data associated with genetic mutations in pathogens and their impact on virulence (Baldwin *et al.*^2006^; Winnenburg *et al.*^2006^; Urban *et al.*2015a,b). At present PHI-base (Version 3.8) comprises 231 pathogenic species including 108 bacterial, 104 fungal, 14 protozoan, 2 aphid and 3 nematode species that infect 143 host species. PHI-base was utilised to identify 112 genetic mutations that caused hypervirulence. The resulting table of 37 pathogen species includes 17 bacterial, 2 protozoan, 17 fungal and 1 nematode species (Fig. 2A; Table S1, Supporting Information). It should be noted that several of the identified pathogens predominantly infect immunocompromised mammalian hosts, which has a profound impact on the pathogen–host interaction. Two pathogenic species stood out in their prominence among the identified hypervirulent genetic mutations, the bacterium *Mycobacterium tuberculosis* and the basidiomycete fungus *Cryptococcus neoformans* (Fig. 2B). Interestingly, both of these pathogens of mammals colonise host macrophages during infection. This occupied intracellular host niche may represent a finely balanced environment that potentially requires mechanisms to evade, manipulate and withstand host immunity, without becoming cytotoxic, which would prevent the establishment of persistent infections.

## THE TRANS-KINGDOM CONSERVED MOLECULAR THEMES AMONG NEGATIVE REGULATORS OF HYPERVIRULENCE

**Figure 3.**
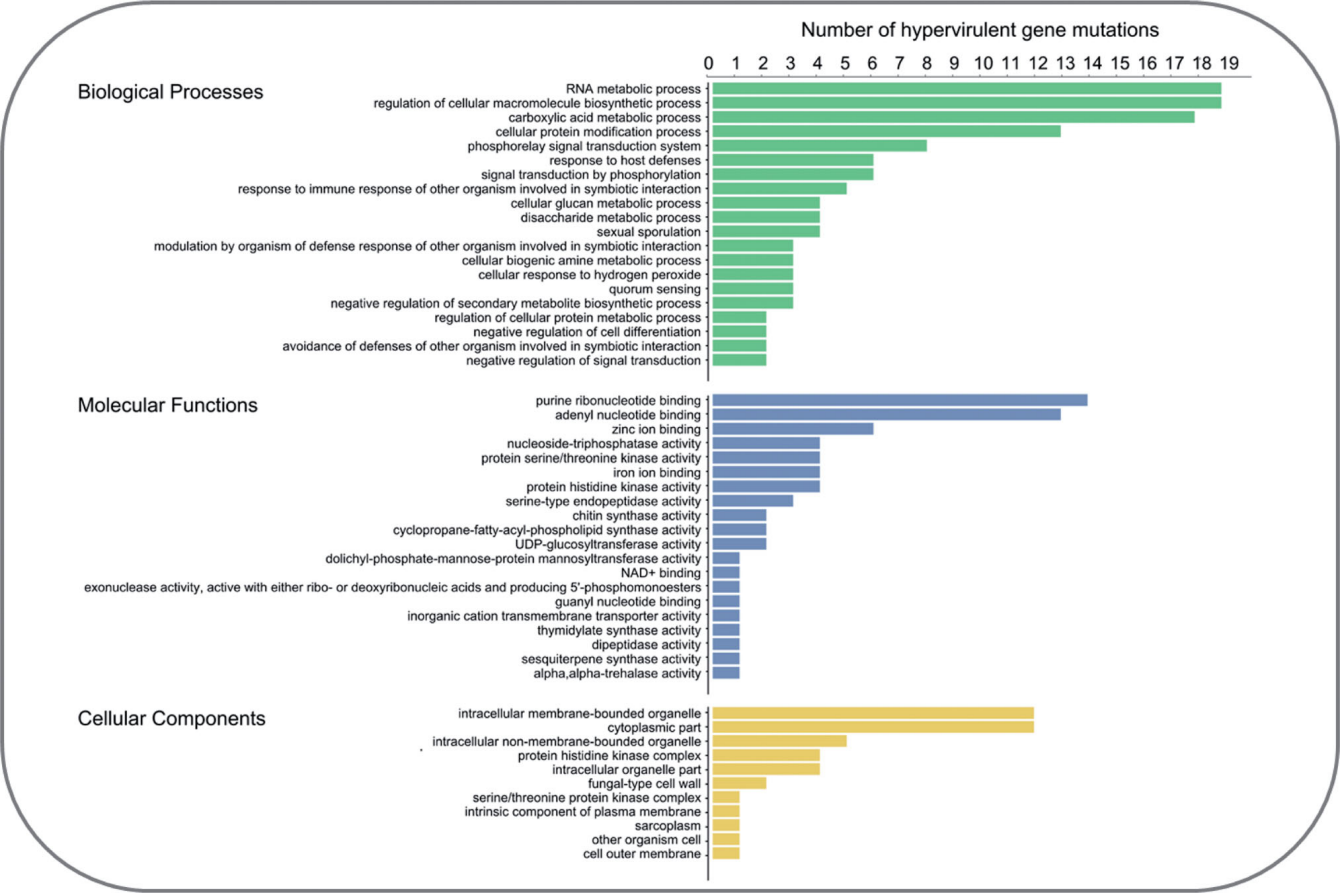
The top 20 biological, molecular and cellular functions of the 112 negative regulators of pathogen hypervirulence. Gene ontologies were identified using the Blast2GO software (Gotz *et al.*^2008^).

The 112 single genetic mutations that caused hypervirulence were interrogated, to reveal the trans-kingdom, conserved, molecular themes among negative regulators of virulence that prevented a hypervirulent interaction. Assessment of the functional annotation of the 112 negative regulators of hypervirulence was performed in Blast2GO and identified the most frequent gene ontologies (Fig. 3). Numerous proteins involved in biological processes that regulate the symbiotic interaction with the host were identified, including the response to immune/defence responses, the modulation of defence responses and the avoidance of defences. Signal transduction systems were also highly represented, including serine/threonine kinases, histidine kinases and quorum-sensing mechanisms. Importantly, multiple negative regulators of signal transduction, secondary metabolite biosynthesis and cell differentiation were revealed. Cellular components and the biosynthetic machinery required for the production of the plasma membrane and the cell wall were also revealed. The conserved molecular and cellular themes among the 112 negative regulators of hypervirulence are discussed using pertinent examples from the different kingdoms of pathogens, with differing host ranges.

### Host perception and how to present one's self appropriately at the ‘court of infection’

Common features of innate immunity in animals and plants include defined pattern recognition receptors (PRR) for microbe- or pathogen-associated molecular patterns (MAMPs or PAMPs), conserved mitogen-associated protein kinase signalling cascades and the production of antimicrobial peptides. These similarities in innate immunity are a consequence of convergent evolution and reflect inherent constraints on how an innate immune system can be constructed (Ausubel 2005; Dodds and Rathjen 2010). Mammalian cells possess membrane bound, predominantly exposed Toll-like (TLR) receptors, which have an extracellular leucine-rich repeat (LRR) and a cytoplasmic TIR protein-interacting domains, and C-type lectin receptors (CLR), while an intracellular TLR-independent system involves nucleotide-binding oligomerisation domain (NOD) proteins (Ausubel 2005; van de Veerdonk *et al.*^2008^). Plant cells also present extracellular LRR-containing and lectin LysM-containing PRRs, in addition to intracellular nucleotide-binding, LRR-containing (NB-LRR) receptors, which structurally resemble mammalian NOD receptors (Wirthmueller, Maqbool and Banfield 2013). The extracellular mammalian TLR, CLR and intracellular NOD receptors detect slowly evolving PAMPs including bacterial lipopolysaccharides, peptidoglycan and flagellin, plus fungal cell wall polysaccharides, chitin and β-1,3 glucans (Ausubel 2005; Dodds and Rathjen 2010). In contrast, the extracellular PRRs of plants detect PAMPs in addition to damage-associated molecular patterns (DAMPs), such as the release of plant cell wall polysaccharides, while the intracellular NB-LRRs respond to pathogen-specific signals such as secreted effectors from bacteria, fungi and protists, which when activated cause a rapid, more potent, response that commonly results in programmed cell death, and immunity/resistance against an adapted pathogen (Jones and Dangl 2006; Wirthmueller, Maqbool and Banfield 2013; Hammond-Kosack and Jones 2015). How a pathogen presents itself during the initial phase of any interaction with its potential host is therefore vital to determining the eventual outcome of events. In turn, genetic mutations in the biosynthetic pathways and regulatory mechanisms that alter the surface of the pathogen, and potentially PAMPs exposure, were highly represented within the hypervirulent mutations, including 8 bacterial and 23 fungal PHI-base entries.

**Figure 4.**
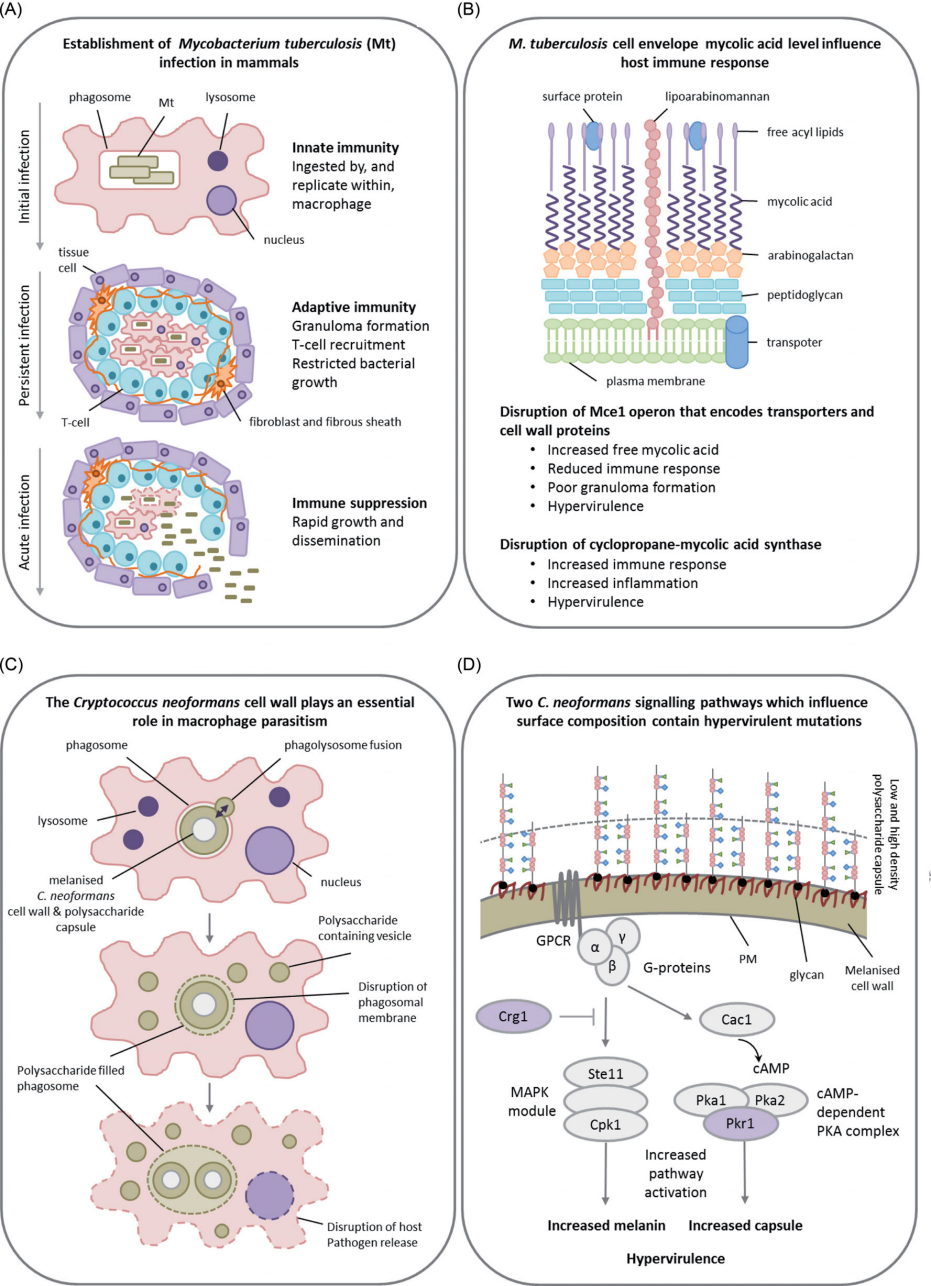
Masking pathogenic potential. Presented are two examples of how the composition of the exterior surface of a bacterial and a fungal pathogen is essential to macrophage colonisation. (**A**) The *M. tuberculosis* infection cycle within a mammalian host, including the establishment of long-term persistent infections via macrophage colonisation. (**B**) The role of the *M. tuberculosis* cell wall in the modulation of host immune responses. Hypervirulence can occur as a consequence of either an elevated or impeded immune response caused by alterations to cell wall composition. (**C**) The fungal polysaccharide capsule and the melanised cell wall of *C. neoformans* promote mammalian infection and macrophage colonisation. (**D**) The absence of negative regulators of the MAPK and cAMP-dependent PKA pathways in *C. neoformans*, which control capsule and melanin biosynthesis, causes hypervirulence. Purple denotes proteins whose absence results in a hypervirulence entry into PHI-base. Abbreviations: PM = Plasma membrane, GPCR = G-protein coupled receptor, Cac1 = Adenylate cyclase, Pka1/2 = PKA catalytic subunits, Pkr1 = PKA regulatory subunit, MAPK = Mitogen-activated protein kinase, Ste11 = MAPKKK, Cpk1 = MAPK, Crg1 = Regulator of G-protein signalling.

The Gram-positive bacterial pathogen *M. tuberculosis* is predicted to infect one third of the world's population causing ∼1.5 million deaths annually (WHO 2015). Important virulence attributes of *M. tuberculosis* include its ability to survive and multiple intracellularly, the induction of the inflammatory response that results in the formation of granulomas, and its ability to enter a dormant state and persist as a latent infection (ten Bokum *et al.*^2008^). Granulomas are a collection of host immune cells that attempt to contain infection. The breakdown of granulomas can release the bacterium into host alveoli, aiding in the transmission of *M. tuberculosis* infections (Fig. 4A). The unusual cell wall of *M. tuberculosis* is rich in lipids, such as the key virulence factor mycolic acid (Meena and Rajni 2010), which reduces the ability of mammalian cells to respond to TLR-2 (Toll-like receptor 2) agonists and induce a proinflammatory response (Sequeira, Senaratne and Riley 2014). Within the lungs of animals, *M. tuberculosis* is taken up by alveolar macrophages. Components of the bacterial cell wall prevent the fusion of the phagosome with a lysosome, but not fusion with nutrient-rich vesicles (Fig. 4A). In addition, *M. tuberculosis* prevents phagosome acidification and neutralises reactive nitrogen intermediates (Meena and Rajni 2010). Collectively, this lipid-rich ‘shield’ presented by the bacterial cell wall enables *M. tuberculosis* to colonise the macrophage. The *M. tuberculosis mce1* (mammalian cell entry 1) operon contains 12 genes, including membrane transporters and surface-exposed cell wall proteins. Disruption of the Mce1 operon results in the increased accumulation of cell wall mycolic acid (Andrea Forrellad *et al.*^2014^), while disruption of two *mce1* genes within this operon, namely, *mce1A* or *yrbE1B*, similarly caused faster replication in mice lungs, hypervirulence, induced poor granuloma formation and a reduced proinflammatory response (Shimono *et al.*^2003^; Joshi *et al.*^2006^). The growth of the *mce1* mutants was attenuated in mixed infections with wild-type bacteria capable of inducing an immune response. Conversely, deletion of the transcriptional repressor of the operon, *mce1R*, leads to the constitutive expression of the operon and also hypervirulence, but via a different mechanism involving an enhanced granulomatous response (Uchida *et al.*^2007^). Additionally, alterations to the biosynthesis of cell envelope lipids, caused by the deletion of the *cmaA2* cyclopropane-mycolic acid synthase which subsequently lacked the *trans*-cylcopropane rings in the cell wall mycolic acid, resulted in accelerated host mortality which was attributed to severe lung lesions caused by an excessive inflammatory response (Rao *et al.*^2006^). Disruption of the putative mycolic acid methyltransferase, *umaA1*, also caused hypervirulence. In wild-type strains, the *umaA1* gene is transcriptionally induced in response to phagocytosis. This suggests that bacterial cell wall remodelling within the macrophage plays a role in hypervirulence (McAdam *et al.*^2002^). Collectively, these results indicate that alterations to the pathogens cell envelope can disrupt the delicate pathological balance resulting in either a weakened or enhanced immune response that accelerates cell death by *M. tuberculosis* (Fig. 4A).

The *Cryptococcus* genus of fungi annually affects approximately one million, predominantly immunocompromised, humans worldwide, causing meningoencephalitis, and accounts for approximately one third of all HIV/AIDS-associated deaths (Byrnes *et al.*^2011^). The facultative, encapsulated, pathogen *C. neoformans* is disseminated from its primary site of infection, the lungs, to the central nervous system, achieving latent, persistent infections (Brown *et al.*^2012^). The production of an extracellular polysaccharide capsule, primarily consisting of glucuronoxylomannans, and melanin are key virulence determinants, enabling macrophage colonisation (Chang and Kwonchung 1994; Salas *et al.*^1996^) (Fig. 4B). Persistent, latent infections in immunocompetent rats are associated with intracellular macrophage colonisation (Goldman *et al.*^2000^). In turn, the absence of biosynthetic enzymes, such as the Cas1 glycosyltransferase required for glucuronoxylomannan *O*-acetylation and the Pmt2 *O*-mannosyltransferase, which caused de-*O*-acetylation, altered capsule formation and resulted in hypervirulence (Janbon *et al.*^2001^; Shimizu *et al.*^2014^). The major intracellular signalling mechanisms, including the cyclic AMP-dependent protein kinase A (PKA), mitogen-activated protein kinase (MAPK), lipid and calcium/calcineurin pathways, regulate capsule formation, melanin biosynthesis and subsequently virulence (Kozubowski, Lee and Heitman 2009). Misregulation of the cAMP-dependent PKA pathway that controls metabolism and growth in response to environmental cues, via the loss of the PKA regulatory subunit, Pkr1, results in elevated pathway induction, the production of enlarged capsules and causes hypervirulence (D'Souza *et al.*^2001^). Additionally, the absence of the negative regulator of G-protein signalling, Crg1, which is involved in desensitising the MAPK-Ste12 pheromone response pathway resulted in increased pathway induction and melanin biosynthesis, causes hypervirulence (Wang *et al.*^2004^). Therefore, in *C. neoformans*, alterations that result in increased capsule or melanin biosynthesis at the surface of the fungal cell can impact upon the host immune response, macrophage colonisation and cause hypervirulence.

During plant pathogenesis, alterations to the exposed surface of bacteria and fungi can also give rise to hypervirulent strains. For example, the Gram-negative bacterium *Xylella fastidiosa* which infects the xylem of plants resulting in blockages and wilting. *X. fastidiosa* causes numerous plant diseases including Pierce's disease of grapevine and Citrus variegated chlorosis disease (Mansfield *et al.*^2012^). In the absence of the Dmt glycosyltransferase, which is potentially involved in cell wall lipopolysaccharide biosynthesis, hypervirulence occurred, which may reflect alterations to cell wall lipopolysaccharide structure (Guilhabert and Kirkpatrick 2005). The filamentous fungus *Alternaria brassicicola* causes black leaf spot on Brassica crops including broccoli, cabbage and canola. This necrotrophic fungal pathogen secretes toxic secondary metabolites and hydrolytic enzymes which induce apoptosis and direct cell damage (Cho *et al.*^2012^). The deletion of the *amr1* transcription factor required for melanin biosynthesis resulted in a melanin-deficient hypervirulent strain (Cho *et al.*^2012^) potentially enhancing PAMP exposure and thus heightening the induction of host cell death. This is in contrast to the previously described *C. neoformans* example where successful fungal colonisation requires live host cells, and elevated melanin biosynthesis successfully shielded against host defences and was associated with hypervirulence (Wang *et al.*^2004^). However, the *amr1* mutation in *A. brassicicola* also results in increased hydrolytic enzyme secretion and the more efficient utilisation of pectin, which may have dually contributed to increased DAMPs-mediated host cell apoptosis and enhanced pathogen fitness.

The filamentous fungal pathogen *Fusarium oxysporum* causes vascular wilt disease on numerous plant species and is also the cause of an emerging disease of immunocompromised mammals (Ortoneda *et al.*^2004^). *F. oxysporum* mutants that lacked the ChsV chitin synthase were unable to infect tomato and were increasingly sensitive to antifungal plant defence compounds. But when tested in a mouse host, hypervirulent, ‘fast-killing’ occurred which was due to the blockage of airways with abnormally large conidia and hyphal swellings. These phenotypes were caused by defects in fungal cell wall biogenesis (Madrid, Di Pietro and Roncero 2003; Ortoneda *et al.*^2004^). This example demonstrates that virulence factors may play functionally distinct roles during the infection of different hosts due to differences in host physiology, pathogen lifestyle and the host immune systems.

Genetic mutations to biosynthetic and/or regulatory pathways that impact upon the composition of the extracellular surface of the pathogen have an impact upon host recognition, while also potentially influencing specialised pathogen mechanisms deployed to modulate or defend against host immunity. These alterations, in turn, can disrupt the balance of pathogenesis and cause hypervirulence.

### Community relationships: working together versus going it alone

Microorganisms have the capacity to grow as either planktonic or sessile cells; however, the former is seldom the case in the natural environment where 80% of microorganisms form social aggregations of cells adhered to a biotic or abiotic surface, termed a biofilm, which permits survival in hostile environments (Hall-Stoodley, Costerton and Stoodley 2004; Ramage and Williams 2013). Microbial biofilms cause 65–80% of human infections, where these heterogenous colonies, encased in an extracellular matrix, are resistant to desiccation, host immune responses, ingestion and antimicrobial agents (Hall-Stoodley, Costerton and Stoodley 2004). The formation of bacterial and fungal biofilms follows the same sequential steps, namely, (1) adherence to a surface, (2) the formation of a colony, (3) secretion of an extracellular matrix, (4) maturation into a 3D structure and (5) cell dispersion (Bonhomme and d'Enfert 2013). The formation of organised colonies requires microorganisms to coordinate efforts via cell-to-cell communication, which subsequently impacts on biofilm formation and other virulence traits such as toxin biosynthesis and reproduction. Quorum sensing (QS) is a cell-to-cell communication mechanism that plays a key role in regulating virulence genes and biofilm formation. Chemical signals released by bacteria or fungi accumulate at high cell densities inducing cell density-dependent transcriptional responses. In bacteria, this is typically mediated by a two-component system (TCS) where the signal interacts with a cognate receptor, to activate a response regulator that controls target gene expression (Solano, Echeverz and Lasa 2014). In fungi, QS involves the detection of oxylipins and pheromones by extracellular G-protein coupled receptors (GPCRs), which regulate the cAMP-dependent PKA pathway that controls gene expression (Mallick and Bennett 2013). It is therefore not surprising to identify 19 bacterial and 6 fungal hypervirulent PHI-base mutations that impact on community structure, cell-to-cell communication, while simultaneously influencing virulence factor production and stress tolerance.

**Figure 5.**
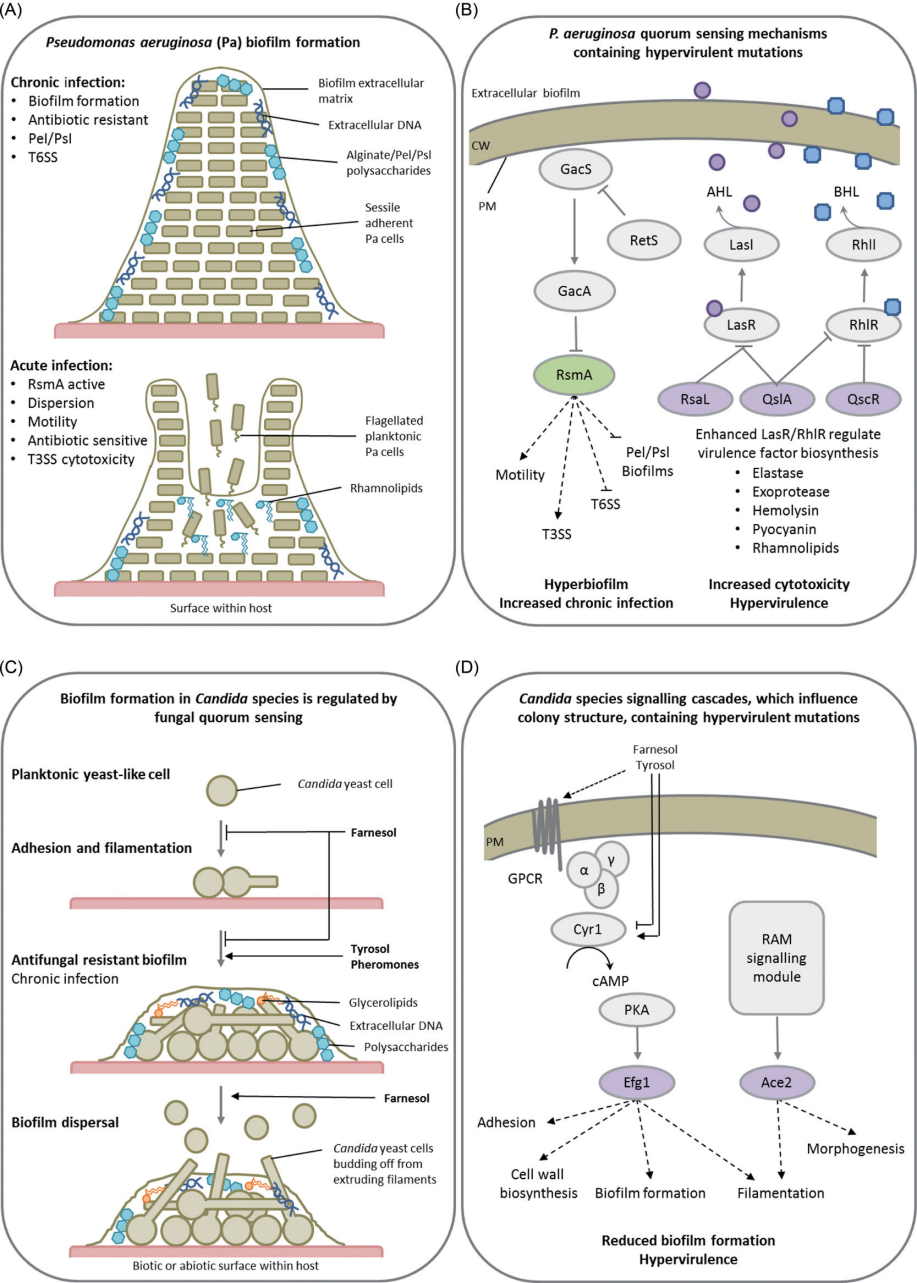
Intercellular communications promote strength in numbers. Presented are two examples of how bacterial and fungal cell-to-cell communication influences community structure and virulence. (**A**) The development of the *P. aeruginosa* bacterial biofilm and the impact of biofilm dispersal in mammalian hosts during the chronic and acute phases of infection. (**B**) Quorum sensing (QS) in *P. aeruginosa* regulates biofilm dispersal and toxin biosynthesis, while the absence of various negative regulators of QS results in hypervirulence. (**C**) QS in *Candida* species regulate fungal morphology in addition to biofilm formation and maturation. (**D**) The absence of two transcription factors, Efg1 or Ace2, regulated by cAMP-dependent PKA and RAM signalling pathways, controls biofilm formation and causes hypervirulence. Purple denotes proteins whose absence results in a hypervirulence entry into PHI-base. Green represents proteins whose absence results in an increase in chronic infection and not hypervirulence. Panel A abbreviations: Pel/Psl = Extracellular polysaccharides, T3SS = Type III secretion system, T6SS = Type VI secretion system. Panel B abbreviations: CW = Cell wall, PM = Plasma membrane, LasR/RhlR = LuxR-type QS receptors, LasI/RhlI = LuxI-type QS synthases, AHL = Acylhomoserine lactone (purple circle), BHL = *N-*butyrylhomoserine lactone (blue square), RsaL/QslA/QscR = QS repressors. Panel D abbreviations: GacS/GacA = Two component system sensor and regulator, RetS = Orphan sensor and repressor of GacS, RsmA = Transcriptional repressor. Panel D abbreviations: GPCR = G-protein coupled receptor, Cyr1 = Adenylate cyclase, PkA = PKA complex, Efg1/Ace2 = Growth regulating transcription factors, RAM = Regulation of Ace2 and morphogenesis pathway.

The Gram-negative bacterium *Pseudomonas aeruginosa* is an opportunistic human pathogen, which causes life-threatening nosocomial infections in immunocompromised humans. *P. aeruginosa* has multiple hierarchically organised QS systems, such as the acylhomoserine lactone (AHL) Las system (homologous to the Lux system in other bacteria), the *N-*butyrylhomoserine lactone (BHL) Rhl system, the 2-heptyl-3-hydroxy-4-quinolone PQS system and the 2-(2-hydroxyphenyl)-thiazole-4-carbaldehyde IQS system (Lee and Zhang 2015). As a consequence, *P. aeruginosa* is known to produce a large number of secreted and cell-associated virulence factors including exotoxins, proteases, phospholipases, siderophores and lipopolysaccharide. The production of these virulence factors is regulated in a cell density-dependent manner by *P. aeruginosa* QS mechanisms (Rumbaugh, Griswold and Hamood 2000). These four *P. aeruginosa* QS systems are required for full virulence (Lee and Zhang 2015). The AHL QS circuits consist of two genes, a LasI-type synthase which produces AHL and a LasR-type AHL-response regulator. However, some *lasR* homologues lack their linked synthase. One such LasR-type orphan, QscR, was shown to govern the timing of AHL-controlled genes and to prevent LasR and RhlR DNA binding, dampening both the Las and Rhl systems, resulting in the higher, and earlier, production of the toxin pyocyanin at lower cell densities (Chugani *et al.*^2001^). LasR also induces the expression of *rsaL*, a transcriptional repressor of *lasI*, which lowers AHL levels (Rampioni *et al.*^2009^). QslA inhibits the LasR protein–protein interaction, raising the AHL-QS threshold (Seet and Zhang 2011) (Fig. 5A). Disruption of any of these QscR, QslA or RsaL negative feedback mechanisms results in increased virulence factor production and hypervirulence (Chugani *et al.*^2001^; Rampioni *et al.*^2009^; Seet and Zhang 2011). Also in *P. aeruginosa*, the GacS/GacA TCS, which is QS dependent, regulates virulence and biofilm formation (Mikkelsen, Sivaneson and Filloux 2011). The response regulator GacA induces two small regulatory RNAs which titrates the transcriptional repressor RsmA, promoting the transcription of genes involved in QS, secretion, cytotoxicity and motility. The absence of RsmA relieved GacA pathway repression, inducing hyperbiofilm formation (Pessi *et al.*^2001^), which in a mouse model of chronic infection resulted in a significantly lower mortality rate in infected mice, yet promoted persistence, resulting in increased murine lung inflammation (Mulcahy *et al.*^2008^). This demonstrates how alterations in TCS and QS regulate the balance between disease severity and persistence, i.e. acute (dispersed) or chronic (persistent, biofilm-forming) infections.

*Vibrio vulnificus* is a Gram-negative bacterium that infects shellfish, which if ingested raw can cause rapid, fatal, septicaemia and high mortality rates in humans (Froelich and Oliver 2013). In *V. vulnificus* the QS repressor, LuxO, promotes *luxT* expression, which negatively regulates the expression of the *smcR* master QS regulator that is required for the transcription of the *vvpE* virulence factor (Roh *et al.*^2006^). The production of the metalloprotease VvpE, by *V. vulnificus*, enhances host vascular permeability and causes haemorrhagic damage, while also being involved in surface adherence/colonisation, by facilitating bacterial swarming, and promoting host invasion by destroying components of mucosal immunity, IgA and lactoferrin (Kim *et al.*^2007^). Deletion of *luxO* resulted in enhanced *smcR* expression, increased metalloprotease production and hypervirulence (Ha *et al.*^2014^). The Gram-negative enteric bacterium, *C. rodentium*, infects mice causing transmissible murine colonic hyperplasia (Mundy *et al.*^2005^) and is used as a comparative model system for the study of significant gastrointestinal pathogens of humans, such as enteropathogenic and enterohaemorrhagic *Escherichia coli* (Collins *et al.*^2014^). *C. rodentium* possesses an AHL-QS mechanism. Deletion of AHL synthase encoding gene, *croI*, in *C. rodentium* resulted in the loss of the QS signal, reduced attachment to abiotic surfaces and caused hypervirulence in mice (Coulthurst *et al.*^2007^). Therefore, in the three aforementioned bacterial pathogens of animals, the AHL-QS mechanisms coordinated virulence factor production, biofilm formation and virulence.

*Erwinia carotovora* subsp. *carotovora* is a Gram-negative bacterium, which causes soft rots on many plants and plant organs, possesses a *N*-(3-oxohexanoyl)-L-homoserine lactone (OHL)-QS system, similar to *P. aeruginosa*, which regulates several virulence determinants including, motility and the secretion of plant cell wall degrading enzymes (PCWDEs) and the proteinaceous toxin, harpin^ECC^ (Pirhonen *et al.*^1993^; Cui *et al.*^1996^). However, in contrast to *P. aeruginosa* where RsmA disruption caused hyperbiofilm formation, disruption of RsmA in *E. carotovora* resulted in the increased expression of flagellar encoding genes, hypermotility, plus increased production of secreted hydrolytic proteins and hypervirulence on celery (Cui *et al.*^1996^). In the potato soft rot causing, Gram-negative bacterium, *Pectobacterium wasabiae*, the orthologous RsmA QS post-translational repressor, also negatively regulates PCWDEs and bacterial cell swarming. Interestingly, the absence of RsmA in *P. wasabiae* resulted in hypervirulence, yet also decreased growth rate and fitness *in vitro*, potentially reflecting the energetic cost of virulence mechanisms (Koiv *et al.*^2013^). In the plant xylem infecting bacterium *X. fastidiosa*, *rpfF* encodes a diffusible signal factor (DSF) synthase involved in the biosynthesis of a QS signalling molecule. Disruption of biosynthetic enzyme, RpfF, resulted in an inability to synthesise XfDSF, impeding QS, altering colony morphology and causing hypervirulence (Ionescu *et al.*^2013^). Intriguingly, the release of outer membrane vesicles that block attachment and biofilm formation, facilitating dispersal throughout the plant via the xylem, was suppressed by QS and derepressed by the disruption of RpfF (Ionescu *et al.*^2014^). Similarly, in *X. fastidiosa*, disruption of two haemagglutinin adhesins, HxfA and HxfB, involved in cell-to-cell aggregations and potentially biofilm formation, caused altered colony morphology and growth rate, in addition to hypervirulence (Guilhabert and Kirkpatrick 2005). Therefore, in bacterial pathogens of plants and animals, QS regulates virulence factor production, adhesion and biofilm formation, while mutations in QS repressor proteins can potentially increase virulence factor secretion and give rise to acute/systemic infections that cause hypervirulence.

*Candida* species are the most common cause of life-threatening invasive fungal infections in immunocompromised patients or those exposed to medical trauma (Brown *et al.*^2012^). Among such pathogens, the dimorphic fungus *Candida albicans* is the species most frequently associated with biofilms, particularly on medical implants, is increasingly azole resistant, and in turn has become a model system for fungal cell-to-cell communication and biofilm formation studies. Initially, *C. albicans* yeast cells adhere to a surface and then develop into a network of filamentous hyphae interspersed with yeast cells covered by an extracellular matrix containing polysaccharides, extracellular DNA and glycerolipids. *C. albicans* genes involved in adherence, matrix formation, QS and morphogenesis have been described as playing a role in biofilm formation (Finkel and Mitchell 2011). In *C. albicans*, key QS molecules, such as farnesol, tyrosol and pheromones, regulate several pathogenic traits including the yeast-to-hyphae transition in a density-dependent manner (Mallick and Bennett 2013). Farnesol inhibits the adenylate cyclase of the cAMP-dependent PKA pathway, in turn inhibiting filamentation and biofilm formation, while possibly also inducing yeast cell formation for dispersal from the biofilm. Tyrosol appears to have an opposing function. Alternatively, pheromone-induced sexual biofilms facilitate a stabilised pheromone gradient and improve mating efficiencies. Large-scale *C. albicans* mutant screens identified multiple transcription factors involved in biofilm formation, including Ace2p of the RAM signalling network and Egf1p downstream of the cAMP-dependent PKA pathway, which regulated morphogenesis, adhesion and cell wall biosynthesis genes (Finkel *et al.*^2012^; Nobile *et al.*^2012^). In *C. albicans*, disruption of Egf1p resulted in hypervirulence (Pierce *et al.*^2013^), while disruption of Ace2p resulted in attenuated virulence (Kelly *et al.*^2004^). Conversely, the disruption of Ace2p in *C. glabrata* resulted in hypervirulence (Kamran *et al.*^2004^). Therefore, in *Candida* species QS also influences morphogenesis, biofilm formation and virulence (Fig. 5B).

The filamentous fungal pathogen *Aspergillus fumigatus* causes a spectrum of clinical diseases in humans, including allergic bronchopulmonary aspergillosis, aspergilloma and invasive aspergillosis (Brown *et al.*^2012^). Biofilms represent an important morphological trait required for the development of aspergilloma, while in bronchopulmonary aspergillosis large hyphal aggregates are formed within the mucus, called mycetoma. In contrast, invasive aspergillosis infections are more diffuse, yet still represented by intertwined hyphal bunches (Ramage *et al.*^2011^). Mature *A. fumigatus* biofilms show reduced metabolic activity, an upregulation of hydrophobins and proteins involved in secondary metabolism, such as gliotoxins (Ramage *et al.*^2011^). Oxygenated polyenoic fatty acids, oxylipins, act as intercellular and potentially interspecies signalling molecules, which in fungi regulate cell growth, differentiation, apoptosis and pathogenicity (Tsitsigiannis and Keller 2007). A collection of oxylipins that regulate both asexual and sexual development are collectively called the psi factor. Three psi-producing oxygenases, namely PpoA-C, contribute to psi factor biosynthesis, fungal development and the biosynthesis of toxic secondary metabolites in *Aspergillus nidulans* (Tsitsigiannis and Keller 2006). A triple *ppo-*silenced *A. fumigatus* strain was hypervirulent in a model of invasive aspergillosis, despite producing the same level of gliotoxin as the parental strain. The increase in virulence was attributed to the moderate increase in fungal growth rate, the reduction in fungal oxylipins, which may serve as activators of immune responses, and the increased resistance to reactive oxygen species, which may protect the pathogen from host defences (Tsitsigiannis *et al.*^2005^). Unfortunately, biofilm formation in the triple *ppo-*silenced *A. fumigatus* strain was not assessed. Nonetheless, oxylipin-mediated cell-to-cell communication negatively regulated virulence and was required for the development of heterogenous, 3D, sexual biofilms.

Cell-to-cell communication in bacteria and fungi controls pathogen growth pattern and the formation of organised heterogeneous communities. The stark contrast between individual planktonic cells and sessile communities is revealed in the differential requirement, and production of secreted virulence factors that are regulated in a cell density-dependent manner by QS mechanisms. The outcome of these contrasting pathogenic strategies is distinct, with dispersed pathogens commonly causing acute infections, while recalcitrant sessile communities promote persistent infections. The division of labour, altered metabolism, efflux mechanism and the extracellular matrix of an organised heterogeneous biofilm create a community that is inherently resistant to host derived, or clinically administered, antimicrobials. In nature, interspecies cell-to-cell signalling is involved in the formation of multiple species communities, influencing pathogenic traits and the virulence outcome. For example, on the skin or within the oral cavity, gastrointestinal and reproductive tracts of humans, *C. albicans* interacts with other microbial species. *P. aeruginosa* is frequently coisolated with hospital-acquired *C. albicans* infections and forms a multiple species biofilm (Mallick and Bennett 2013). The *P. aeruginosa* QS molecules and virulence factors can suppress *C. albicans* yeast-to-hyphal transition, while limiting its growth, biofilm formation and decreasing virulence (Morales *et al.*^2013^). Similarly, the bacterium and biocontrol agent *Pseudomonas chlororaphis* can produce a phenazine-1-carboxamide antifungal that suppresses tomato foot or root rot caused by the soil-borne fungus *F. oxysporum* f. sp. *radicis-lycopersici*. However, the fungus can secrete fusaric acid which alters bacterial QS mechanisms, inhibiting phenazine-1-carboxamide production and permitting fungal infections of tomato (van Rij *et al.*^2005^). Intriguingly, such mixed microbial interactions do not only represent competition between pathogens, as the bacterium *Klebsiella aerogenes* provides precursors for the biosynthesis melanin by *C. neoformans*, which is a key virulence determinant for the fungal pathogen (Frases *et al.*^2006^). Therefore, intra- and interspecies cell-to-cell signalling can determine the sessile or planktonic growth pattern adopted by a pathogen, which is coordinated with virulence factor production, can potentially influence the switch between a commensal and pathogenic interaction or increase stress resistance with the host organism. Hence, the evolution, formation and dispersal from these pathogenic communities have a profound impact upon virulence, the treatment of infection and the potential rise of hypervirulence.

### Maintaining the pathogenic balance: morphogenesis and growth rate

Pathogens deploy specialised morphogenic programmes and modify their growth rates during pathogenesis. These shifts in growth pattern promote and maintain the evolutionary optimised pathogenic balance within each host. A total of 40 PHI-base mutations, including 16 bacterial, 21 fungal, 2 protozoan and 1 nematode PHI-base entries, altered the coordination of pathogen growth, disrupting this balance with the host organism, causing hypervirulence.

Tuberculosis rarely results from the progression of primary *M. tuberculosis* infections and normally occurs after the reactivation of latent infections. Granulomas are organised structures of immune cells that restrict microbial infections (Fig. 4A). *M. tuberculosis* can persist within granulomas, growing slowly, for decades (Guirado and Schlesinger 2013). Misregulation of the balance between pro- and anti-inflammatory responses can lead to bacterial reactivation, granuloma breakdown and tuberculosis (Fig. 4A, lower panel). *M. tuberculosis* contains 11 complete TCS and several unlinked sensor kinase or response regulator homologues (Cole *et al.*^1998^). In *M. tuberculosis*, different TCS have been shown to regulate pathogenicity and growth rate. For example, the DosR/DevR regulator controls the expression of ∼50 genes, including the 80-fold induction of the heat shock protein encoding gene *hspX*, in response to environmental cues such as hypoxia and nitric oxide (Hu *et al.*^2006^). Disruption of DosR/DevR or HspX resulted in increased growth *in vivo* within mice and *in vitro* within both resting and activated macrophages, causing hypervirulence. The overexpression of *hspX* in the DosR/DevR mutation background restored wild-type growth rates and virulence (Hu *et al.*^2006^). Intriguingly, HspX was shown to be the major membrane protein that accumulated in the cell wall (Cunningham and Spreadbury 1998). Therefore, HspX appears to possess additional functions beyond acting as a cytosolic chaperone. HspX is speculated to play a structural role in the *M. tuberculosis* cell wall which directly, or indirectly, affects the growth rate of the bacterium. Similarly, disruption of three additional TCS-QS mechanisms in *M. tuberculosis*, TcrXY, TrcS and KdpDE, also caused increased growth rates and hypervirulence (Parish *et al.*^2003^). Therefore, restricting growth within macrophages may play an essential role in the establishment of latent infections, while disruption of these growth-restricting mechanisms can result in hypervirulence.

*Salmonella enterica* serovar Typhimurium causes self-limiting gastroenteritis in humans. This enteric bacterium survives within macrophages and utilises them to be disseminated from the intestine to the spleen and liver (Mastroeni and Grant 2013). The outcome of the interaction with the macrophage depends on two type III secretion systems, namely SPI-1 which is involved in cell invasion, promoting intestinal infection, and SPI-2 which enables intracellular macrophage survival and systemic infection. Both systems are required for full virulence and both have the capacity to induce macrophage cell death by different mechanisms (Hueffer and Galan 2004). Additionally, a type IV secretion system gene cluster is also required for host cell infection. However, deletion of a single gene within this cluster, *sciS*, resulted in enhanced growth within macrophages and hypervirulence (Parsons and Heffron 2005). The TCS regulator, SsrB, activates the SPI-2 pathogenicity island and repressed *sciS* expression, promoting intracellular survival, limiting bacterial overgrowth and maintaining the pathogenic balance.

**Figure 6.**
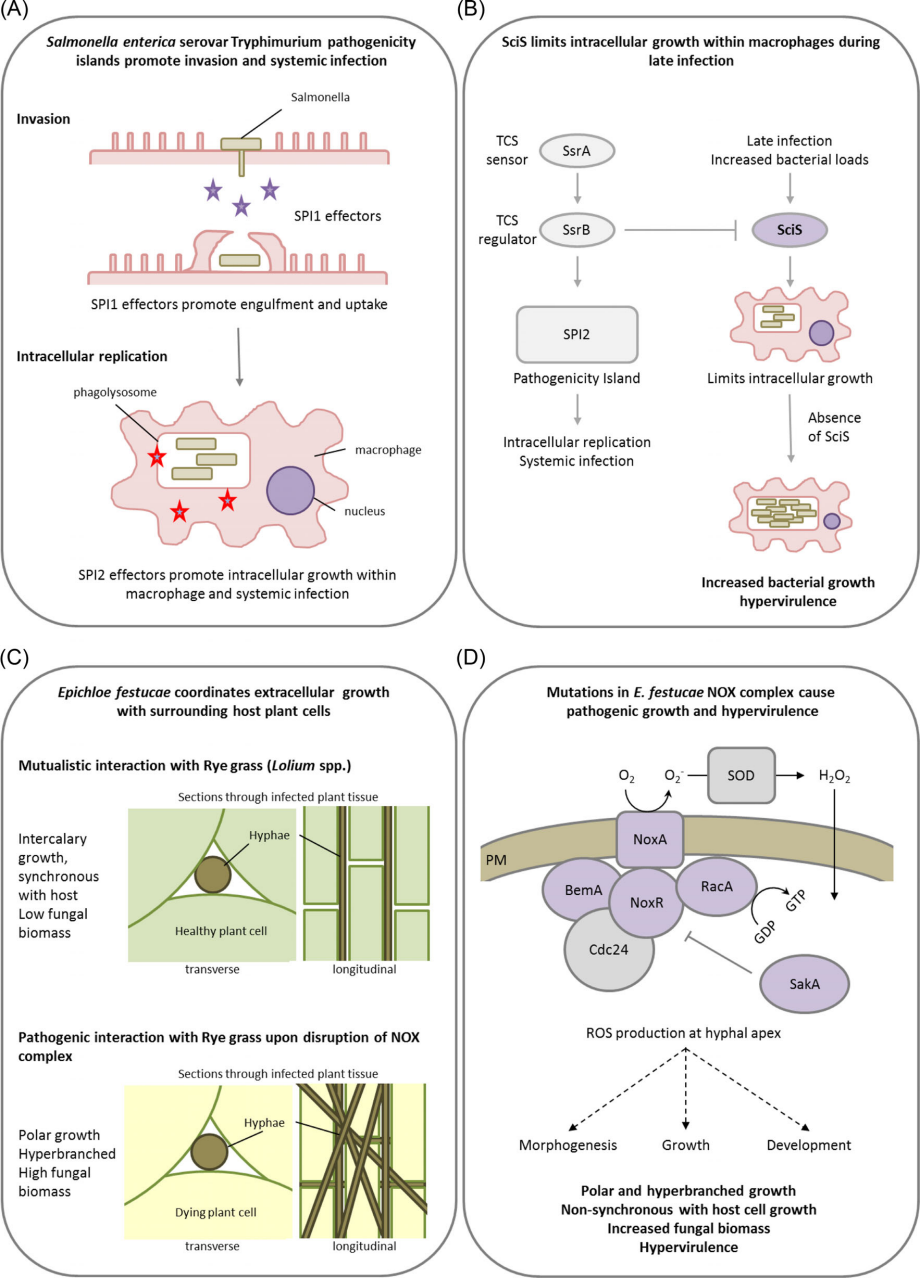
The pathogenic balance: morphogenesis and growth rate. Presented are two examples of how a bacterial and a fungal pathogen restrict pathogenic morphogenesis and growth to maintain a highly adapted intracellular or a tightly coordinated intercellular form of infection. (**A**) *Salmonella enterica* serovar Tryphimurium pathogenicity islands promote mammalian host invasion, intracellular macrophage colonisation and systemic infection. (**B**) SciS limits intracellular growth within macrophages during late infection and the absence of SciS causes increased intracellular replication and hypervirulence. (**C**) In the fungus, *E. festucae*, the NOX complex and related signalling pathways coordinate mutualistic grass colonisation and prevent pathogenicity. (**D**) The absence of a single NOX component, excluding Cdc24, causes increased ROS production, pathogenic hyphal growth, and results in hypervirulence. Purple denotes proteins whose absence results in a hypervirulence entry into PHI-base. Panels A and B abbreviations: SPI1 and SPI2 = Samonella pathogenicity island 1 and 2, SsrA = TCS sensor, SsrB = TCS regulator, SciS = Salmonella centisome 7 island. Panel D abbreviations: BemA = Scaffold protein involved in cell polarity and orthologue of *Saccharomyces cerevisiae* Bem1, NoxA = NADPH oxidase, NoxR = p67phox-like regulator, RacA = Small GTPases, Cdc24 = Guanine nucleotide exchange factor, SakA = Stress-activated MAPK, SOD = Superoxide dismutase, ROS = Reactive oxygen species.

Plant symbiotic fungi form intimate associations with host cells whether in a pathogenic or mutualistic interaction. *Epichloe festucae* forms a mutualistic association with perennial ryegrass (*Lolium perenne*). This association benefits the fungus by providing access to nutrients and a means of dissemination, while the host benefits from increased resistance to herbivory through the production of toxic fungal alkaloids, which impacts on grazing livestock. *E. festucae* synchronises its intercalary hyphal growth with the surrounding elongating leaf cells to maintain a tightly regulated symbiotic state (Christensen *et al.*^2008^). An active NADPH oxidase (NOX) complex consisting of NoxA, NoxR, RacA, BemA and Cdc24 is required at sites of fungal morphogenesis and growth to maintain the mutualistic interaction (Takemoto, Tanaka and Scott 2006; Tanaka *et al.*^2006^, ^2008^; Takemoto *et al.*^2011^). Excluding Cdc24, disruption of any of the other NOX complex components resulted in hyperbranched hyphal growth within ryegrass leaves, which no longer aligned parallel to the leaf axis as well as increased fungal biomass resulting in the breakdown of a mutualistic interaction and the development of pathogenesis (Fig. 6B). In addition, disruption of two of the most important signal transduction pathways for responding to environmental cues, the stress-activated (SakA) and the cell wall integrity (MkkA and MpkA) MAPK pathways also caused the switch from restricted to proliferative pathogenic growth, while also altering additional virulence traits such as hydrolytic enzyme production and chitin exposure (Eaton *et al.*^2010^; Becker *et al.*^2015^). Therefore, mutations in signalling pathway that delicately balance morphogenic programmes coordinating fungal growth within a host can cause hypervirulence.

G-protein signalling in eukaryotic cells plays an essential role in conveying extracellular signals into intracellular signal transduction pathways. In fungi, G-proteins and the cAMP-dependent PKA pathway regulate vegetative growth, morphogenesis, metabolism and pathogenicity (Bolker 1998; Lengeler *et al.*^2000^; Yu 2006). Crucial for the correct functionality and sensitisation of these signalling cascades are negative feedback mechanisms such as the repressors of G-protein signalling (RGS) and the cAMP phosphodiesterases. The filamentous ascomycete *Magnaporthe oryzae* causes devastating rice and wheat diseases threatening global food security. *M. oryzae* produces specialised infection structures called appressoria to penetrate the plant surface, permitting rice blast disease (Xu and Hamer 1996). The appressorium forms at the tip of a germ tube in response to specific environmental and physicochemical surface cues and is regulated by G-protein signalling and the cAMP-dependent PKA pathway. In *M. oryzae*, the repressors of G-protein signalling, Rgs1, and the phosphodiesterase PdeH modulate conidiogenesis, hydrophobic surface signalling and pathogenesis (Liu *et al.*^2007^; Ramanujam and Naqvi 2010). Disruption of either Rgs1 or PdeH resulted in precocious appressorial development on non-inductive surfaces. Despite increasing penetration of the host plant, the disruption of PdeH resulted in reduced pathogenesis. Similarly, a T-DNA insertion *Leptosphaeria maculans* mutant, which causes Phoma stem canker disease on Brassica crops, demonstrated an increase in the frequency of penetration of the plant *Arabidopsis thaliana* but did not confer an overall higher level of disease (Elliott, Harjono and Howlett 2008). Therefore, mutations that result in increased initial plant host penetration can actually be detrimental to overall pathogen virulence.

Pathogens that form intimate associations with host cells, such as those which colonise live host cells or tissue, restrain proliferative growth within the host to maintain the pathogenic balance and to avoid inducing host cell death. This pathogenic balance has evolved to aid the epidemiological strategy of the pathogen. Pathogenic bacteria, protozoa and fungi have subsequently acquired genetic mechanisms to limit motility and growth, promoting the spread of disease. In turn, mutations that impeded these self-restricting mechanisms caused increased pathogen motility, via enhanced growth rate, resulting in hypervirulence. However, this uncoupling of the delicately balanced interaction between pathogen and host may be counterproductive to the overall epidemiology of the disease. Future alteration to the pressures exerted upon the pathogen, such as climate change, environmental stress, host resistance mechanisms and human intervention, could shift this balance and promote the evolution of hypervirulence, when increased pathogen motility becomes epidemiologically beneficial.

### Manipulating the host's response: the secretion of toxins, effectors and hydrolytic enzymes

Interspecies communication is paramount in determining the outcome of the intimate symbioses represented by host–pathogen interactions. Pathogens secrete numerous toxic and non-toxic metabolites, proteinaceous effectors and nucleic acids that promote infection. Due to differences in virulence strategies, pathogens utilise the secretion of such virulence determinants to avoid host recognition, manipulate host responses, induce host cell death and liberate nutrition (Brown and Hammond-Kosack 2015). However, the biosynthesis of such compounds comes with an energetic cost, and an optimal balance must be established to prevent detrimental side effects on virulence and pathogen fitness. Within the hypervirulence gene list, a total of 33 PHI-base mutations, including 17 bacterial and 16 fungal PHI-base entries, altered virulence factor biosynthesis and secretion.

**Figure 7.**
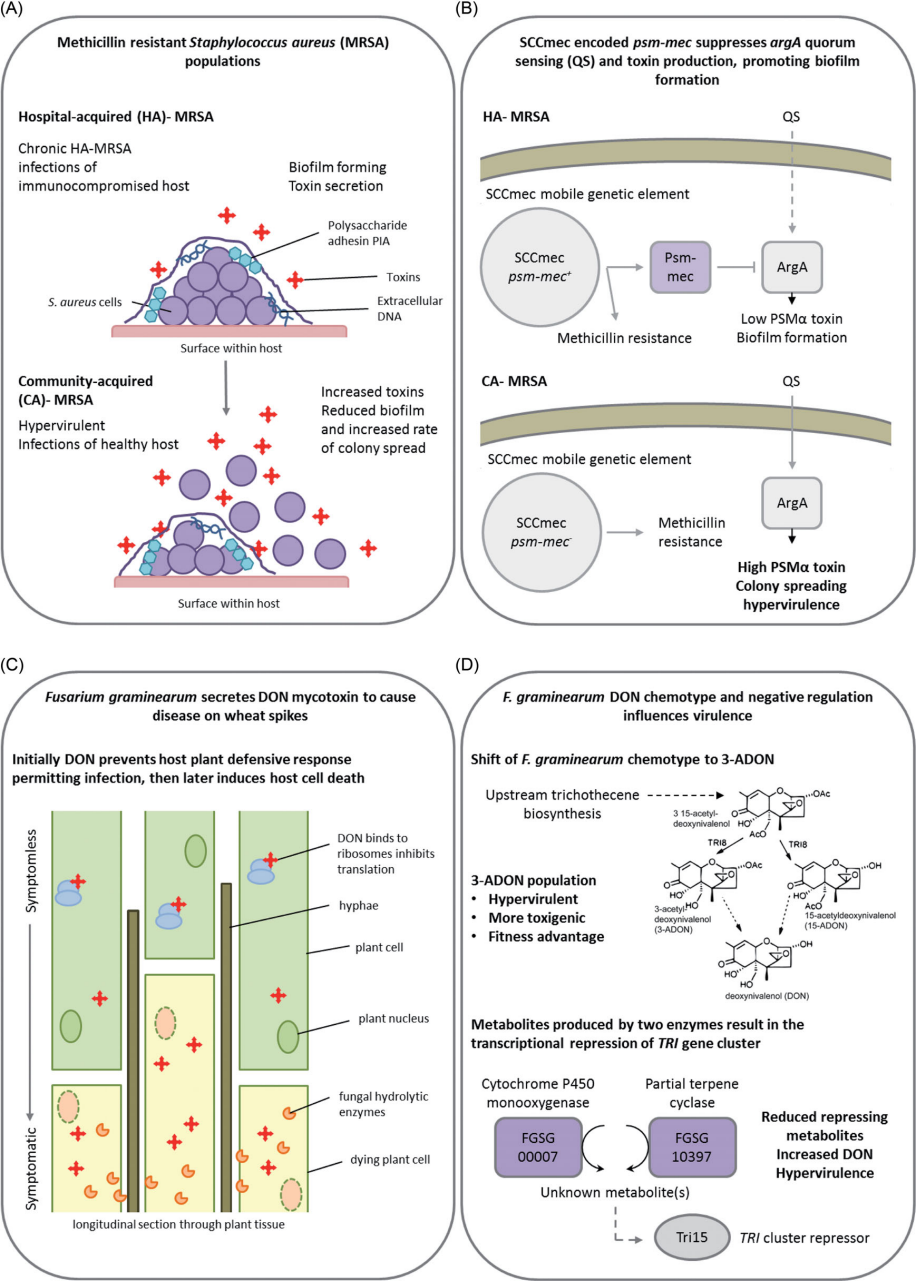
Negative regulators of toxin biosynthesis. Presented are two examples of how alterations to the regulation and biosynthesis of secreted virulence factors can give rise to hypervirulence in a bacterial and a fungal pathogen. (**A**) The contrasting profiles of hospital (HA)- and community (CA)-acquired methicillin resistant *Staphylococcus aureus* (MRSA). (**B**) A single gene on a mobile genetic element accounts for differing virulence profile of HA-MRSA and CA-MRSA. The *psm-mec* gene encoded on the SCC*mec* mobile genetic element suppresses AgrA-mediated quorum sensing and attenuates virulence in HA-MRSA, while the absence of *psm-mec* in CA-MRSA results in hypervirulence. (**C**) *Fusarium graminearum* secreted a trichothecene mycotoxin, DON to inhibit host plant defence responses promoting infection. (**D**) The natural emergence of a hypervirulent 3A-DON chemotype with increased fitness. In addition, the absence of a cytochrome P450 monooxygenase or a partial terpene cyclase results in the transcriptional derepression of DON mycotoxin biosynthesis, causing increased DON secretion and hypervirulence. Purple denotes proteins whose absence results in a hypervirulence entry into PHI-base. Panel A abbreviation: PIA = Polysaccharide intercellular adhesin. Panel B abbreviations: QS = Quorum sensing, ArgA = Response regulator of the Arg two component system. Panel D abbreviations: *TRI* = Gene encoding protein involved in trichothecene biosynthesis, Tri15 = Transcriptional repressor of *TRI* gene cluster.

Toxins are detrimental/poisonous substances that directly interfere with the host and are distinct from other virulence factors which potentiate disease through host manipulation. The secretion of toxins by pathogens is a highly conserved virulence strategy. *Staphylococcus aureus* is a versatile Gram-positive bacterial pathogen that can cause a multitude of human diseases such as life-threatening nosocomial infections, allergenic reactions and symptom-free colonisation (Otto 2014). *S. aureus* secretes numerous toxins including haemolysins, leukotoxins and the phenol-soluble modulins (PSMs). PSMs contribute to neutrophil lysis after phagocytosis, which is of immense importance to infection, in addition to roles in the disruption of the extracellular matrix and biofilm dispersal (Le *et al.*^2014^; Otto 2014). Increased toxin production directly correlated with enhanced *S. aureus* virulence and the AgrA-mediated QS mechanism, which promotes toxin production and biofilm dispersal. A mobile genetic element, SCC*mec*, confers resistance to the antibiotic, methicillin, and contains the *psm-mec* gene, which if introduced to methicillin-sensitive strains lacking SCC*mec* suppresses colony-spreading ability, promotes biofilm formation and decreases PSMα secretion, via suppressing the translation of the QS regulator ArgA. Disruption of *psm-mec* in methicillin-resistant clinical strains caused increased AgrA translation, higher PSMα secretion and hypervirulence in mouse skin and systemic infection models (Kaito *et al.*^2013^) (Fig. 7A).

*A. nidulans* is an important saprophytic mould and a model system for the study of fungal cell biology, while invasive *A. nidulans* pulmonary infections are common in humans with chronic granulomatous disease (Henriet, Verweij and Warris 2012). In conjunction with the VeA velvet protein, the nuclear localised methyltransferase, LaeA, acts as a global regulator of secondary metabolism. Disruption of *laeA* blocked the expression of metabolic gene clusters, including the sterigmatocystin, penicillin and lovastatin pathways (Bayram *et al.*^2008^). Fungi possess additional LaeA-like methyltransferases. In *A. nidulans*, a LaeA-like methyltransferases, LlmF, opposes the role of LaeA and acts as a negative regulator of sterigmatocystin production via interacting directly with VeA (Palmer *et al.*^2013^). However, the impact on virulence in immunocompromised patients is unknown. In other fungal pathosystems, the orthologous mechanism has been demonstrated to be involved in virulence. The necrotrophic fungal pathogen, *Cochliobolus heterostrophus*, exists as two races, race O and race T, with the latter producing T-toxin and causing Southern Corn Leaf Blight on maize. The nine genes associated with T-toxin biosynthesis are only found in race T, are not organised as a cluster of genes, do not share a common evolutionary history and are fragmented across two chromosomes (Inderbitzin, Asvarak and Turgeon 2010). Nonetheless, the methyltransferase Lae1 and velvet protein Vel1 positively regulate T-toxin production in response to light, and therefore impact upon virulence (Wu *et al.*^2012^). As in *A. nidulans*, an Lae1-like methyltransferases, Llm1, negatively regulates T-toxin production in *C. heterostrophus* and the disruption of Llm1 resulted in increased maize leaf chlorosis, while *llm1* overexpression had the opposite effect (Bi, Zhu and Turgeon 2013).

The hemibiotrophic cereal infecting fungal pathogen, *Fusarium graminearum*, causes direct grain yield and quality losses, in addition to contaminating the grain with trichothecene toxins, such as deoxynivalenol (DON), which have severe consequences for human and animal health. In wheat spikes, DON production is upregulated during symptomless infection, where it prevents protein translation and host responses to infection (Cuzick, Urban and Hammond-Kosack 2008; Brown *et al.*, 2010; Brown *et al.*^2011^). The trichothecene biosynthetic enzyme encoding genes are clustered together in several genomic loci, which also include two *tri* gene inducers, *tri6* and *tri10*, plus a putative *tri* gene repressor, *tri15* (Kimura *et al.*^2007^). Two genes beyond the *tri* cluster, which encode a cytochrome P450 monooxygenase (FGSG_00007) and a partial terpene cyclase (FGSG_10397), are coordinately regulated with the *tri* genes in a *tri6-*dependent manner. Disruption of either gene resulted in increased *tri* gene expression, elevated DON production and hypervirulence on wheat (Gardiner, Kazan and Manners 2009) suggesting that these genes are responsible for the production of metabolites that negatively regulate DON at the transcriptional level, potentially mediated by Tri15 (Fig. 7B). In agricultural settings, *F. graminearum* subpopulations can be divided according to the type of DON mycotoxin produced. Emerging 3-acetyl-DON populations in North America are more resilient to temperature fluctuations, and produce more infective spores and DON, than the original 15-acetyl-DON population. The outcome is an overall increase in virulence (Ward *et al.*^2008^; Puri and Zhong 2010) (Fig. 7B) which is potentially contributing to the switch of the agriculture-associated population back to a predominantly 3-acetyl-DON chemotype. Similarly, emerging Asian *F. graminearum* and *F. asiaticum* 3-acetyl-DON populations are more vigorous and toxigenic than the 15-acetyl-DON and nivalenol populations, in addition to being more fungicide resistance (Zhang *et al.*^2012^). The causes underlying these population level shifts in DON chemotype and the rise of the more aggressive *Fusarium* 3-acetyl-DON populations are unknown. However, DNA marker analyses have revealed substantial genetic differences (Puri and Zhong 2010). Collectively, these examples reveal that in bacteria and fungi, mutations in genes that negatively regulate toxin production will potentially give rise to pathogens with increased virulence. But at what fitness costs to the pathogen and how do these hypervirulent pathogen variants produce more toxin without causing self-harm?

Current understanding of the evolution of proteinaceous effectors is most advanced in the study of plant–pathogen interactions where effectors, which are secreted by a pathogen to counteract host innate immunity and promote infection, have been shown to drive the coevolution of specialised host mechanisms for pathogen recognition. This direct or indirect interaction between pathogen effectors and their cognate host resistance (*R*) proteins/‘guardee’ proteins activates host defence mechanism, controlling host resistance (Stergiopoulos and de Wit 2009). An effector recognised by the host is referred to as an avirulence (*Avr*) gene, due to the induction of host resistance. The loss or mutation of an *Avr* gene can result in increased virulence on host genotypes with the corresponding functional *R* protein. In monogenic agricultural crop species, this absence of plant resistance conferred by cognate *Avr* and *R* proteins frequently leads to severe disease epidemics and yield losses.

The basidiomycete fungal pathogen, *Ustilago maydis*, infects maize plants, resulting in stunted plant growth and the formation of tumours on the maize cob. This biotrophic interaction is represented by the intracellular colonisation of host cells, which requires the secretion of numerous effectors to promote infection and prime the host cell for colonisation, without inducing detrimental host defensive responses and host cell death (Kaemper *et al.*^2006^; Djamei *et al.*^2011^). The *U. maydis* genome encodes 12 gene clusters for effector proteins which are involved in virulence. The absence of individual effector clusters predominantly resulted in attenuated virulence. However, the Um01234–41 effector cluster was atypical, where disruption of the entire cluster resulted in hypervirulence (Kaemper *et al.*^2006^). The genes within this cluster may therefore encode weak *Avr* proteins that trigger defence responses in their plant host carrying the cognate receptor. Hence, the absence of *Avr* proteins can negate host defence mechanisms, enhance pathogenic growth and cause hypervirulence.

Numerous plants infecting bacteria and fungi secrete hydrolytic enzymes to breakdown the host cell in order to gain access, induce host cell death and acquire nutrition (Van Sluys *et al.*^2002^; Brown and Hammond-Kosack 2015). The secretion of hydrolytic enzymes in bacteria and fungi is tightly controlled by carbon catabolite repression (CCR) which is regulated by a complex network of environmental-sensing mechanisms (Gorke and Stulke 2008; Brown, Ries and Goldman 2014). Quorum- and carbon-sensing mechanisms coincide in the activation of cAMP signalling pathways. Therefore, disruption of quorum and carbon sensing can result in the deregulation of CCR and elevated hydrolytic enzyme production, causing hypervirulence. This is the case for disruptions of RsmA in the bacteria *E. carotovora* subsp. *carotovora* and *P. wasabiae*, plus the glucokinase responsible for the formation of the CCR-inducing compound that initiates intracellular hexose signalling in *X. fastidiosa*. Similarly, disruption of the CCR-repressor protein, CcpA, in *Streptococcus pyogenes* and *P. aeruginosa*, resulted in increased toxin production and hypervirulence (Kinkel and McIver 2008; Zhang *et al.*^2013^). The disruption of other environmental-sensing mechanisms that are known to influence CCR in fungi, such as the *F. oxysporum* PacC-negative regulator of acid-expressed genes (Caracuel *et al.*^2003^) and the *E. festucae* stress-activated MAPK, SakA (Eaton *et al.*^2010^), also caused increased hydrolytic enzyme secretion and hypervirulence. In *A. brassicicola* the disruption of Amr1-mediated transcription, which is required for melanin biosynthesis, also derepressed hydrolytic enzyme production, facilitated pectin utilisation and caused hypervirulence, suggesting that Arm1 was important for saprophytic survival yet negatively influenced virulence (Cho *et al.*^2012^).

Pathogens withstand the substantial energetic burden of secreting toxins, effectors and hydrolytic enzymes to promote infection, creating the need for, and evolution of, mechanisms to repress such traits when not required. During infection, pathogens respond to environmental stimuli including, nutrient availability and cell-to-cell signalling, by coordinating the correct spatio-temporal biosynthesis of toxins, effectors and hydrolytic enzymes, via the aforementioned transcriptional mechanisms. All these different classes of secreted virulence factors are only beneficial to the pathogen under specific conditions. Mutations in genes involved in such repressing mechanisms will therefore commonly lead to hypervirulence. However, increased virulence factor production may potentially come at a cost to pathogen fitness and disease epidemiology, due to their potential to self-harm, the increased energetic burden and the rapid induction of host death prior to dissemination.

### Miscellaneous negative regulators of hypervirulence

Not all of the 112 hypervirulent PHI-base mutations, which are representative of negative regulators of pathogen hypervirulence, fit into the aforementioned functional categories. Currently, only 16 PHI-base mutations did not fit into the identified trans-kingdom themes for the negative regulation of hypervirulence previously described. Nonetheless, these 16 PHI-base entries are of equal merit and several require discussion. The IpgB1 effector secreted by the Gram-negative bacterium, *Shigella flexneri*, which causes gastroenteritis and dysentery in animals, promotes bacterial entry through the ELMO-Dock180 machinery (Hachani *et al.*^2008^). The absence of IpgB1 causes hypervirulence, potentially via influencing bacteria adherence, community structure and/or virulence factor delivery into the host cells. The chorismate mutase enzyme is found in bacteria, fungi and higher plants, where it catalyses a key reaction in the shikimate pathway involved in the biogenesis of amino acids such as tyrosine and phenylalanine. The absence of chorismate mutase, *AroQγ*, in the Gram-negative bacterial pathogen and causal agent of rice leaf blight, *Xanthomonas oryzae* pv. *oryzae*, resulted in hypervirulence (Degrassi *et al.*^2010^). Interestingly, the fungal pathogen, *U. maydis*, and several root-knot nematodes secrete a chorismate mutase into the host cell as a mechanism to prime host metabolism for subsequent infection (Bekal, Niblack and Lambert 2003; Doyle and Lambert 2003; Djamei *et al.*^2011^), demonstrating the diversity of functions for a single enzyme in pathogenesis. Copper is a redox-active transition metal that has functions in enzymatic reactions, while excess copper can result in the production of damaging hydroxyl radicals. Therefore, transition metal homeostasis is tightly regulated in host cells. Invading pathogens compete for the limited transition metals in order to colonise and replicate during infection (Schaible and Kaufmann 2004). Guinea pigs (*Cavia porcellus*) respond to *M. tuberculosis* infection by increasing the copper concentration in the lungs, while the absence of the mycobacterial copper transport protein, MctB, which is essential for copper tolerance, resulted in attenuated virulence (Wolschendorf *et al.*^2011^). In contrast, the absence of the Ctr4 copper transporter in the fungal pathogen *C. neoformans* caused hypervirulence and an increased inflammatory response in mice (Sun *et al.*^2014^). Overall, these 16 non-classified hypervirulent PHI-base mutations may represent species-specific biological functions and/or unidentified unifying themes which will become apparent as the documentation of the hypervirulent phenotype expands.

## THE EVOLUTIONARY SIGNIFICANCE, AND TRANS-KINGDOM REQUIREMENT, FOR NEGATIVE REGULATORS OF HYPERVIRULENCE

Pathogens and pests from the bacterial, protozoa, fungal and animal kingdoms have demonstrated the convergent evolution of single genetic elements that regulate virulence via altering host perception, morphology, community structure and/or the secretion of virulence determinants. The energetic burden of undertaking morphological adaptations and the biosynthesis of virulence factors may have a fitness penalty on the pathogen, while pathogen-derived inhibitory, hydrolytic and toxic compounds may have the potential to self-harm. These costs must be counteracted by an increase in the pathogen's ability to reproduce on the host and the dissemination of the disease (Fig 1.).

Community structure, in particular biofilms, influences the exposure of the microorganism to pathogen-derived (i.e. toxins) or host-derived (e.g. antimicrobials) detrimental compounds. Compact, slow growing, recalcitrant colonies can withstand higher levels of exposure to toxins or antimicrobials, permitting the establishment of persistent infections, while dispersed rapidly growing pathogens appear more destructive. However, in nature pathogens rarely adopt a dispersed growth pattern during the establishment of infection (Van Acker, Van Dijck and Coenye 2014). Instead, pathogens predominantly interact with others, forming biofilms, specialised infection structure, swarming or undergoing sexual/asexual recombination to promote infection. From this established situation, dispersed growth forms can facilitate pathogen dissemination. It is these persistent infections and their inherent resistance mechanisms involving a shielding extracellular matrix, efflux pumps and detoxifying enzymes (Van Acker, Van Dijck and Coenye 2014) that have facilitated the evolution of antimicrobial resistance, whether that be within a host or in a clinical, agricultural or industrial setting.

To be a successful pathogen, an evolutionary balance between the morphological adaptation and the biosynthesis of virulence factors with their potential detrimental impacts on pathogen fitness and their beneficial influence on pathogenesis or antimicrobial resistance must be established. This balance is maintained by the opposing action of pathway-specific positive and negative regulators. It is these virulence phenotypes determining transcriptional, translational and biosynthetic steps that are subject to evolutionary pressure that aligns pathogenic potential with the host environment. Accordingly, PHI-base catalogued 14 and 10 hypervirulent mutations which were negative regulators of toxin biosynthesis or hydrolytic enzyme secretion, respectively. These mutations tip the balance to favour the pathogen. This highlights the evolutionary requirement to restrict harmful levels of toxin production and the expenditure of energy on unrequired hydrolytic enzymes. While 20 and 31 hypervirulent PHI-base mutations were negative regulators of cell-to-cell communication and the establishment of biofilms or growth patterns which are tightly coordinated with the host, respectively.

## PHI-base MUTATIONS LINKED TO NATURAL HYPERVIRULENCE AND DISEASE OUTBREAKS

Emerging infectious diseases (EIDs) pose an increasing threat to human health, agricultural productivity and food security, in addition to natural ecosystems (Jones *et al.*^2008^). EIDs are defined as (1) an increase in incidence (geographic or host range), (2) changed pathogenesis and (3) newly evolved or discovered diseases (Daszak, Cunningham and Hyatt 2000). Anthropogenic influences on land use, agrochemical usage, antimicrobial drug treatments and the international movement of people and commodities have shifted the composition and structure of natural and man-made ecosystems (Table 1). Of particular concern is the rise of hospital- and community-acquired pathogen resistance to antimicrobials, driven by the use of drugs with a common mode of action in agriculture, livestock and humans (Cohen 2000). Global climate change is also influencing the geographic distribution of hosts and pathogens, while also dually influencing the capacity of the pathogen to cause disease and the host's ability to mount a defensive response (Table 1). Climate changes have facilitated the gradual northward polar movement of fungal and bacterial pathogens to maintain their temperature optima (Bebber *et al.*^2013^). Therefore, anthropogenic and climatic factors have a profound impact on the evolution and distribution of EIDs. The hidden cost of human economic development may be the enhanced evolution of EIDs driven by anthropogenic factors.

**Table 1.** Environmental factors behind naturally occurring disease outbreaks.

Factor	Trait	Cause	Examples	Reference
Anthropogenic influences	Hospital-acquired resistance	Increased antimicrobial drug usage; increased disease transmission	Methicillin-resistant *Staphylococcus aureas*; Azole-resistant *C. albicans*	Cohen (2000)
	Community-acquired resistance	Agricultural antimicrobial usage	Multidrug-resistant *Salmonella*; Triazole-resistant *A. fumigatus* and *Rhynchosporium commune*	Cohen (2000); Verweij *et al.* (2009); Hawkins *et al.* (2014)
	Global transport of infected hosts	Redistribution	*Cryptococcus gatti*-eucalyptus; *Batrachochytrium dendrobatidis*­amphibians; *Phytophthora ramorum-*ornamental plants	Fisher *et al.* (2012); Taylor and Gurr (2014)
Climate change	Altered geographic distribution of pathogen and host	Warmer winters and higher humidity increase pathogen build-up; earlier start of infection cycle; storm dispersal	Pathogens move polewards; re-emergence of Fusarium Ear blight in North America	Fisher *et al.* (2012); Bebber *et al.* (2013)
	Extreme weather	Example: El Niño years cause a rise in sea temperatures	*Vibrio parahamolyticus* infection of shell-fish causing outbreak in food poisoning	Cohen (2000)
	Droughts	Can dually reduce pathogen infectivity, yet increase host susceptibility	*Aspergillus flavus* infections of maize and Aflatoxin contamination	Mauch-Mani and Mauch (2005); Anderson *et al.* (2004)

**Figure 8.**
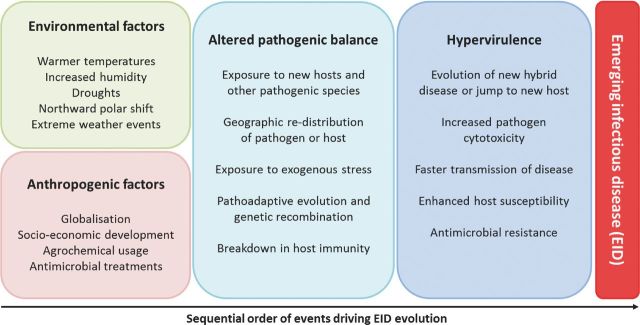
Anthropogenic and environmental factors driving the evolution of emerging infectious diseases. Specific anthropogenic and environmental factors are increasingly having an impact on interactions between pathogen and host, driving rapid evolutionary changes within the pathogen that can give rise to a hypervirulence phenotype, new diseases, altered host range and/or antimicrobial resistance. Subsequently, the same anthropogenic and environmental factors can facilitate the dissemination and rise in prevalence of the newly adapted pathogens, representing the increasing risk of EIDs, threatening human and animal health, agriculture and natural ecosystems.

These anthropogenic and climatic factors are projected to increase the generation of new diseases by bringing together pathogens and hosts that may result in new hybrid species and/or an altered host range (Fig. 8), as demonstrated by the cereal fungal rust epidemics (Helfer 2014). Additionally, altered environmental pressures exerted on a pathogen–host interaction impacts upon the pathogenic balance, driving pathoadaptive evolution and potentially giving rise to highly adapted and hypervirulent pathogens. Therefore, two evolutionary models dominate in the explanation for the differences in virulence profile between naturally occurring strains of the same species: (1) the acquisition of virulence genes from other organisms, and (2) pathoadaptive mutations that alter the regulation and/or the function of existing genes. The former model is not yet specifically curated and represented within PHI-base and has been extensively reviewed elsewhere (Ochman, Lawrence and Groisman 2000; Koonin, Makarova and Aravind 2001; Gogarten, Doolittle and Lawrence 2002; Oliver and Solomon 2008; Mehrabi *et al.*^2011^; Gardiner *et al.*^2012^; Soanes and Richards 2014). These anthropogenic or climatic factors may influence the maintenance, mutation or loss of negative regulators of virulence, by shifting the costs and benefits of pathogenesis, potentially driving the rise of hypervirulence. The adaptive evolutionary potential of the pathogen is usually greater than the host, due to their shorter generation times and often haploid genomes. Either via pathoadaptive evolution or genetic recombination and/or the acquisition of new virulence genes, pathogens may rapidly evolve mechanisms to cause disease on new hosts, within new territories and under new climatic conditions, outpacing the capacity of the hosts to adapt to the conditions and the new pathogenic burden. Hence, the frequency of repeated and unpredictable disease outbreaks, with the potential to cause host extinction events, is therefore predicted to increase (Fisher *et al.*^2012^; Bebber *et al.*^2013^).

The correlation between laboratory-derived data, such as that predominantly retained within PHI-base, and data obtained from natural occurring pathogen–host interactions may always be disputed. Nonetheless, numerous similarities exist between PHI-base gene mutations and the determinants of disease outbreaks (Table 2). Multiple cases of natural mutations in QS mechanisms and negative regulators of toxin production have been identified in hypervirulent pathogenic bacteria associated with disease outbreaks. For example, the bacterium *S. aureus* is the most common causal agent of hospital-acquired infections associated with high mortality rates (Otto 2014). Antibiotic resistance is a major clinical challenge including hospital-acquired methicillin-resistant *S. aureus* (HA-MRSA) infections of immunocompromised humans. However, the rise of community-acquired methicillin-resistant *S. aureus* (CA-MRSA), which has increased virulence and the capacity to infect healthy humans, represents a greater threat to society (Fig. 7A). The SCC*mec* mobile genetic element of clinical CA-MRSA strains does not contain the *psm-mec* gene and lacks the ability to inhibit PSMα production and colony spreading, promoting biofilm formation (Kaito *et al.*^2013^). HA-MRSA strains predominantly carry the SCC*mec*-encoded *psm-mec* gene, while mutations to *psm-mec* can result in higher PSMα toxin production. Therefore, *psm*-*mec* acts as a negative regulator of ArgA-mediated QS that inhibits toxin production, while acting as a genetic determinant for high virulence (Kaito *et al.*^2013^). Additionally, whole genome analyses of an MRSA strain carrying the SCC*mec* element, associated with both community outbreaks and hospital-acquired infection, identified the insertion of a transposable element into the promoter of the master regulator of virulence factors and repressor of toxins, Rot, resulting in the derepression of toxin biosynthesis and increased virulence (Benson *et al.*^2014^). Hence, the rise of persistent antimicrobial-resistant *S. aureus* infections could have laid the foundations for the evolution of hypervirulent antimicrobial resistant infections, which represents a far greater threat. Additionally, the acquisition of the genetic loci that confer the ability to produce host-selective toxins by new races of two fungal pathogens, *Cochliobolus heterostrophus* and *Pyrenophora tritici-repentis*, gave rise to significant disease outbreaks in maize and wheat, respectively (Ciuffetti, Tuori and Gaventa 1997; Baker *et al.*^2006^). Therefore, the genetic and phenotypic data collected within PHI-base can be utilised to predict the genetic signatures that may be associated with a rise of hypervirulence within a natural population, facilitating the monitoring of EID development.

**Table 2.** Naturally occurring genetic mutations contributing to disease outbreaks reported within PHI-base.

Pathogen	Natural hypervirulent mutation	Function	Effect of mutation on virulence traits	Reference
*Streptococcus pyogenes* Group A	CsrRS(CovRS)	Quorum sensing	Increased streptolysin O production	Sumby *et al.* (2006); Ato *et al.* (2008)
	FasBCAX	Quorum sensing	Increased streptokinase and GRAB	Cao *et al.* (2014)
	Rgg(RopB)	Negative regulator of virulence genes	Increased streptolysin O	Ikebe *et al.* (2010)
*Staphylococcus aureus*	Psm-mec	Quorum sensing	Increased PSMα	Kaito *et al.* (2013)
	Rot	Negative regulator of toxins	Increased toxins	Benson *et al.* (2014)
*Clostridium difficile*	TcdC	Negative regulator of toxins	Increased toxins	Carter *et al.* (2011)
*Shigella flexneri*	CadAB	Lysine decarboxylase	Increased enterotoxin activity	Maurelli *et al.* (1998)
Entero-hemorrhagic, aggregative, pathogenic, invasive and toxigenic *E. coli*	CadAB	Lysine decarboxylase	Increased enterotoxin activity; increased cell adhesion	Torres *et al.* (2005); Vazquez-Juarez *et al.* (2008); Hwang *et al.* (2010)
*Cochliobolus heterostrophus*	PKS1/PKS2	Toxin	Gain of host-selective toxin; altered host range	Baker *et al.* (2006)
*Pyrenophora tritici-repentis*	ToxA	Toxin	Gain of host-selective toxin; altered host range	Ciuffetti *et al.* (1997)

## EXPLORING HYPERVIRULENCE—DIFFICULTIES AND OPPORTUNITIES

An increased understanding of a pathogen's biology, negative regulators of hypervirulence, evolution, environmental factors and anthropogenic influences are needed to predict, prevent and combat the rise of EIDs. Inherent difficulties in the study of uncontrolled natural events and large-scale epidemiological data have hampered the identification of the driving forces behind EID evolution and their impact on society. Governmental licence regulations understandably restrict the creation and study of hypervirulent pathogens of animals and plants. Despite the artificial generation of hypervirulent pathogens presenting ethical safety issues, the availability of near isogenic strains with and without the hypervirulence trait provides the opportunity to truly define the contribution of single genetic elements in the evolution of EIDs. Specialised containment facilities are required to enable the artificial generation and study of hypervirulent pathogen evolution in a secure setting. The hypervirulence data within PHI-base came from 37 pathogenic species. Therefore, authorised protocols and containment facilities are already in place for handling and long-term storage of these precise genetic resources.

Modern experimental approaches to tackle the issue of EID evolution, such as the serial passage of pathogens through constant interaction with a host, followed by high-throughput genome sequencing, will facilitate the identification of a cohort of genetic elements that can potentially give rise to hypervirulence. For example, in *Candida* species microevolution experiments have rapidly resulted in the creation of strains of increased virulence. Cocultivation of *C. glabrata* for six months with murine macrophages resulted in a striking alteration to fungal morphology, switching from yeast-like to pseudohyphal growth, alterations in the fungal cell wall, and hypervirulence. This was shown to be the result of a single nucleotide point mutation within the *chs2* chitin synthase involved in cell wall biosynthesis (Brunke *et al.*^2014^). Similarly, the *C. albicans cph1Δ/efg1Δ* mutant, which is unable to undergo the transition into filamentous hyphae, recovered filamentous growth post 19 cycles of cocultivation with macrophages (Wartenberg *et al.*^2014^). Therefore, persistent exposure of a pathogen to the host environment can rapidly induce the evolution of, or rise in prevalence of, mutations that promote the survival of, and escape from, host defences by causing pathoadaptive mutations and increased virulence.

The combination of prolonged pathogen–host cocultivation experiments with exogenous stresses representative of the aforementioned anthropogenic and climatic influences, such as antimicrobial drugs, rising carbon dioxide levels and/or elevated temperatures, will assist in forecasting the mechanisms of pathoadaptive evolution and the threat of hypervirulence. The amalgamation of this genetic and phenotypic data in an open access resource, such as PHI-base, will in turn aid the scientific community in their study of EIDs. These controlled laboratory experimental strategies to replicate the evolution of EIDs will therefore allow the development and assessment of novel techniques in mitigating EIDs, safeguarding human and animal health, food security and global ecosystems.

Nonetheless, the threat of EIDs will never be completely abolished. Resources such as the Program for Monitoring Emerging Diseases (ProMed) and the HealthMap (www.promedmail.org; www.healthmap.org) which document and monitor EID outbreaks, and CABI which records historical plant disease data and distribution maps (www.cabi.org) are also required to evaluate trends in the natural evolution of EIDs (Anderson *et al.*^2004^; Fisher *et al.*^2012^). Targeted monitoring of natural EID hotspots and at-risk host species will enable the rapid identification of new, naturally occurring EIDs. Monitoring the occurrence of mutations to negative regulators of hypervirulence in natural agricultural/clinical pathogen populations, as identified by reverse genetic experimentation and recorded in PHI-base, as well as the arrival of hybrid EIDs species through pathogen recombination or the introduction of new hosts into pathogen diversity hotspots, as noted in ProMed, HealthMap and CABI, will provide the opportunity to tackle adapted pathogen variants before their large-scale redistribution and rise to prevalence. This smart surveillance of the natural arrival of new pathogen species, plus antimicrobial resistance and hypervirulent variants of existing pathogenic species, will therefore help to mitigate the impact of EID on plant and animal health when changes arise.

Combining global population genetics of pathogen and host, climatic and EID distribution data with an enhanced understanding of the molecular determinants of hypervirulence will permit the development of predictive models for how, and where, new EIDs will evolve, assisting in the allocation of resources designed to prevent and control these new biotic threats. This will require an integrated interdisciplinary approach, and long-term commitments, from the academic, industry and political communities alike to combat the present, forever changing, and increasing threat from pathogens to the health of urban and rural societies, agricultural crops, farmed animals and natural ecosystems.

## FUTURE PERSPECTIVE

Our scientific understanding of the natural rise of hypervirulent clinical, agricultural or environmental pathogens is hampered by the detection of multiple genetic differences that may account for altered virulence. The developmental reprogramming, the production of virulence factors and the increased stress of embarking on a pathogenic interaction are stringently regulated to reduce the metabolic burden on a pathogen. A pathogenic optimum has evolved to balance the cost and rewards of pathogenesis to maintain the fitness of the pathogen, while promoting pathogen dispersal.

This review of the currently known suite of negative regulators of pathogen hypervirulence, encoded by single genetic elements, highlights the evolutionary significance of negative regulators of hypervirulence, while revealing how the current environmental situation is encouraging the emergence of natural mutation in these regulatory mechanisms, giving rise to increased disease and/or pathogen dissemination. In our efforts to combat pathogens in agricultural and clinical settings through chemical intervention (Andersson and Hughes 2010; Cools and Hammond-Kosack 2013) are we subsequently driving the evolution of hypervirulence? Our expanding direct and indirect knowledge of how negative regulators of hypervirulence balance the costs and benefits of pathogenicity will facilitate our understanding of how human activities are contributing to the evolution of EIDs. This new knowledge will enable smart surveillance of pathogen population for genetic signs of a rise in hypervirulence within hotspots for EID emergence. By gradually identifying the drivers of EID evolution, processes to mitigate the spread of hypervirulence pathogens can be devised and implemented.

## Supplementary Material

Supplementary Data
